# Effect of stocking density on behavioral traits, blood biochemical parameters and immune responses in meat ducks exposed to heat stress

**DOI:** 10.5194/aab-61-425-2018

**Published:** 2018-10-30

**Authors:** Byung-Sung Park, Kyung-Hwan Um, Sang-O Park, Victor A. Zammit

**Affiliations:** 1 College of Animal Life Science, Kangwon National University, Chuncheon, Gangwondo, 200-701, South Korea; 2 Metabolic Biochemistry, Warwick Medical School, University of Warwick, Coventry CV4 7AL, UK

## Abstract

High stocking density (HSD) and heat stress (HS) caused by climate change can lower blood homeostasis and negatively impact the behavioral traits of animals. The objective of this study was to explore the influence of stocking densities on behavioral traits, blood parameters, immune responses, and stress hormones in meat ducks (Cherry valley, *Anas platyrhynchos*) exposed to HS. A total of 320 meat ducks were assigned to four groups with different stocking densities using a randomized complete block design. The ducks were then reared for 42 days. The assigned density groups were as follows: (1) control group (CON, three birds m${}^{-2}$, normal environmental heat conditions); (2) low stocking density (LSD, three birds m${}^{-2}$, heat stress conditions); (3) medium stocking density (MSD, four birds m${}^{-2}$, heat stress conditions); and (4) high stocking density (HSD, six birds m${}^{-2}$, heat stress conditions). To induce HS, the environment of the poultry house was set to a temperature of $34\pm 2{}^{\circ }$C with a relative humidity of 70 % from 11:00 to 16:00 for the finisher period (from day 22 to day 42 of the rearing period). Concentrations of blood triacylglycerol, total cholesterol, low-density lipoprotein cholesterol (LDL-C), aspartate aminotransferase (AST), and alanine aminotransferase (ALT) were higher in the HS groups compared with the CON group, with HSD showing the highest levels (P<0.05). The concentrations of high-density lipoprotein cholesterol (HDL-C) and glucose were lower in the HSD groups than in the CON group (P<0.05). Red blood cell (RBC) and platelet (PLT) counts were lower in HS groups compared with the CON group, with the HSD group displaying the lowest counts (P<0.05). Blood pH values were also higher in the HS groups than in the CON group, with the highest values observed in the HSD group (P<0.05). Concentrations of blood p${\mathrm{CO}}_{2}$, ${\mathrm{HCO}}_{3}$, and T${\mathrm{CO}}_{2}$ were higher in the HS groups than in the CON group, with HSD showing the lowest levels (P<0.05). The concentration of ${\mathrm{PO}}_{2}$ was higher in CON than in any of the HS groups, with the lowest levels found in the HSD group (P<0.05). The concentrations of blood IgG and corticosterone were increased in the HS groups compared with the CON group (P<0.05). Animal behavioral trait scores were also higher in HS groups than in the CON group (P<0.05); these scores were the highest in the HSD group. Overall, animal behavioral traits, blood biochemical parameters, and immune responses in meat ducks exposed to heat stress were highest in the HSD group, but not significantly different between the LSD or MSD groups.

## 1 Introduction

Heat stress (HS) and high stocking density (HSD) are known to negatively impact the behavioral traits and growth performance of animals while simultaneously increasing health problems and mortality (Daramola et al., 2012; Slimen, 2016). As birds, including ducks, are covered with feathers and do not have sweat glands, when they are exposed to high temperatures of around 41${}^{\circ }$C under HS and HSD conditions, their body temperatures continue to rise to levels that can damage homeostasis (Etches et al., 2008; Mello et al., 2015).

Another potential and pivotal issue regarding the rearing environment of poultry, particularly meat ducks, is the stocking density, which is known to affect animal behavioral traits. As the number of birds per space unit increases, economic gains can potentially be obtained. However, a high stocking density can be detrimental to animal growth performance, health, and behavioral traits and can lead to more serious damage (Xie et al., 2014). Therefore, increasing the stocking density as a way of boosting earnings has the potential to reduce productivity, especially when birds are exposed to HS during the summer (Chen et al., 2015). As the behavioral traits of animals can change as a result of changes in the rearing environment, the rearing field of economic animals has began to regulate the stocking density. The permissible stocking density for meat ducks in the USA is seven birds m${}^{-2}$ (HSUS, 2008). At stocking densities of more than nine birds m${}^{-2}$, the final body weights and the body weight gains of birds are decreased (Xie et al., 2014). In Korea, the optimal stocking density of meat ducks without any observable changes in the animal's behavioral traits is set at 10.2 kg (three birds) m${}^{-2}$.

HSD rearing can cause a number of problems, including damaging animal health as a result of negative impacts on behavioral traits. For birds under HS during the summer, the higher the stocking density, the lower their behavioral trait score with issues such as footpad dermatitis, scratches, bruising, and poorer feathering having been observed (Na et al, 2012; Xie et al., 2014). Although there have been many studies focused on the effect of HS and stocking density on the productivity of broiler chickens (Yu et al., 2007; An et al., 2012), few such studies have been carried out on meat ducks. As HS in summer seasons can affect the nutritive condition and the activation of metabolism which affects homeostasis in birds, it is important to measure the blood biochemical parameters of the animals as biomarkers of their health (Aengwanich, 2007; Alaeldein, 2013; Habibu et al., 2014). These parameters have been used as general criteria for judging HS and the immunity of poultry (Habibu et al., 2014). In birds, including meat ducks, HS and HSD rearing can increase the excretion of potassium and sodium via fecal urine, which, in turn, can lower electrolytes and change the acid–base balance, the osmotic pressure, and the cell membrane potential (Bang et al., 2015). Poultry exposed to HS and HSD conditions are vulnerable to blood alkalosis caused by panting which can decrease blood gases such as p${\mathrm{CO}}_{2}$ and increase blood pH (Borges et al., 2003, 2004). However, little is known about the effect of stocking density on factors such as the blood corticosterone level, the heterophil (H) : lymphocyte (L) ratio, and the duration of tonic immobility (TI duration) of meat ducks exposed to HS and HSD in the summertime (Altan et al., 2000; Turkyilmaz, 2008; Babacanoglu et al., 2013). Therefore, the objective of this study was to explore the influence of stocking density on behavioral traits, blood biochemical and hematological parameters, immunoglobulin G, and corticosterone levels of meat ducks exposed to heat stress.

## 2 Materials and methods

### 2.1 Animals and experimental design

This study, which used laboratory animals, was approved by the Institutional Animal Care and Use Committee (IACUC) of Kangwon National University (approval no. 2013-0017). The experiment was conducted in accordance with regulations set forth in the European laboratory animal care licensing criteria (ScotPIL training manual, 1994). At the date of incubation, 320 male meat ducks were chosen and divided into grower periods (from day 1 to day 21) and finisher periods (from day 22 to day 42) for the purpose of rearing. HS was maintained in a poultry house where the environment was set to $34\pm 2{}^{\circ }$C and a relative humidity of 70 % from 11:00 to 16:00 for the finisher period (from day 22 to day 42). The birds were randomly divided into four groups: (1) the control group (CON, three birds m${}^{-2}$, normal environmental heat conditions); (2) low stocking density (LSD, three birds m${}^{-2}$, heat stress conditions); (3) medium stocking density (MSD, four birds m${}^{-2}$, heat stress conditions); and (4) high stocking density (HSD, six birds m${}^{-2}$, heat stress conditions). Each stocking density experiment was repeated on three different occasions. Commercial diets and experimental diets were divided and differentially fed to subject birds during grower periods (metabolizable energy, ME 2900 kcal kg${}^{-1}$, crude protein 22 %) and finisher periods (ME 3000 kcal kg${}^{-1}$, crude protein 18 %).

### 2.2 Slaughter and blood sampling

At the end of the experiment, 15 meat ducks per treatment group, which were representative of the average weight of the group, were chosen and blood samples were taken. Euthanasia was then stably administered via cervical dislocation; the animals were not stressed, in accordance with the recommendations for the euthanasia of laboratory animals (Close et al., 1997). Next, 5 mL of blood was collected from the wing vein of each meat duck and kept at room temperature for 15 min prior to centrifugation at 1008 g in order to obtain serum samples. To analyze blood biochemical parameters, serum samples were rapidly frozen using $-196{}^{\circ }$C liquid nitrogen and then stored at $-20{}^{\circ }$C until the next measurement.

**Table 1:** Effect of different stocking densities on the blood lipid profiles and levels of glucose, AST, and ALT in meat ducks exposed to heat stress.

	Treatment groups			
Items${}^{1}$	CON	LSD	MSD	HSD
TC (mg dL${}^{-1}$)	$250.1\pm 6.35^{2,c}$	$314.8\pm 7.51^{2,b}$	$316.5\pm 2.54^{b}$	$348.9\pm 1.85^{a}$
HDL-C (mg dL${}^{-1}$)	$81.07\pm 2.11^{a}$	$76.07\pm 1.30^{a}$	$80.50\pm 0.99^{a}$	$53.51\pm 1.64^{b}$
TAG (mg dL${}^{-1}$)	$167.5\pm 3.27^{c}$	$185.9\pm 2.70^{b}$	$187.2\pm 3.42^{b}$	$205.9\pm 2.83^{a}$
LDL-C (mg dL${}^{-1}$)	155.3${}^{b}$ ± 3.54${}^{c}$	179.6 ± 2.24${}^{b}$	176.5 ± 2.20${}^{b}$	206.0 ± 3.46${}^{a}$
Glucose (mg dL${}^{-1}$)	186.0 ± 3.55${}^{a}$	166.0 ± 3.20${}^{a}$	151.1 ± 2.05${}^{b}$	153.0 ± 2.03${}^{b}$
AST (IU L${}^{-1}$)	52.05 ± 4.13${}^{c}$	61.22 ± 1.35${}^{b}$	60.99 ± 1.08${}^{b}$	75.71 ± 1.42${}^{a}$
ALT (IU L${}^{-1}$)	50.17 ± 2.01${}^{c}$	56.56 ± 0.73${}^{b}$	55.59 ± 1.07${}^{b}$	66.04 ± 1.28${}^{a}$

${}^{1}$ TC represents total cholesterol, HDL-C represents high-density lipoprotein cholesterol, TAG represents triacylglycerol, LDL-C represents low-density lipoprotein cholesterol, AST represents aspartate aminotransferase, and ALT represents alanine transferase. ${}^{2}$ Mean values ± standard errors (n=15). ${}^{a,b,c}$ represent significant differences among the treatment groups at a confidence level of 95 % (P<0.05).

### 2.3 Determination of biochemical parameters

Triacylglycerol (TAG), total cholesterol (TC), low-density lipoprotein cholesterol (LDL-C), high-density lipoprotein cholesterol (HDL-C), glucose, alanine transferase (ALT), and aspartate aminotransferase (AST) levels were analyzed using a precision microplate reader (Molecular Devices Inc., New York, USA) following the protocol from the biochemical assay kit (Sigma-Aldrich, Co. Ltd., USA).

**Table 2:** Effect of different stocking densities on red blood cell and platelet profiles of meat ducks exposed to heat stress.

	Treatment groups			
Items${}^{1}$	CON	LSD	MSD	HSD
RBC				
HCT (%)	37.52 ± 0.91${}^{2,a}$	34.62 ± 0.78${}^{b}$	33.22 ± 0.62${}^{b}$	30.59 ± 0.41${}^{c}$
RBC (M µL${}^{-1}$)	4.83 ± 0.23${}^{a}$	2.30 ± 0.02${}^{b}$	2.31 ± 0.07${}^{b}$	1.93 ± 0.07${}^{c}$
MCV (fL)	152.3 ± 1.65${}^{a}$	145.6 ± 1.73${}^{b}$	142.8 ± 1.65${}^{b}$	139.6 ± 0.88${}^{c}$
MCH (pg)	50.13 ± 0.64${}^{a}$	46.23 ± 1.88${}^{b}$	46.37 ± 0.64${}^{b}$	39.22 ± 0.47${}^{c}$
MCHC (g dL${}^{-1}$)	35.43 ± 0.23${}^{a}$	31.57 ± 0.38${}^{b}$	32.57 ± 0.12${}^{b}$	29.18 ± 0.49${}^{c}$
RDW (%)	8.12 ± 0.35${}^{a}$	7.60 ± 0.25${}^{b}$	7.07 ± 0.15${}^{b}$	4.64 ± 0.38${}^{c}$
HGB (g dL${}^{-1}$)	13.38 ± 0.28${}^{a}$	11.32 ± 0.33${}^{b}$	10.70 ± 0.17${}^{b}$	9.22 ± 0.39${}^{c}$
Platelets				
PLT (K µL${}^{-1}$)	0.41 ± 0.05${}^{a}$	0.27 ± 0.03${}^{b}$	0.26 ± 0.04${}^{b}$	0.11 ± 0.02${}^{c}$
PCT (%)	0.50 ± 0.02${}^{a}$	0.36 ± 0.08${}^{b}$	0.26 ± 0.04${}^{b}$	0.12 ± 0.03${}^{c}$
MPV (fL)	8.58 ± 0.17${}^{a}$	7.52 ± 0.41${}^{b}$	6.83 ± 0.20${}^{b}$	3.81 ± 0.15${}^{c}$

${}^{1}$ HCT represents hematocrit, RBC represents red blood cell, MCV represents mean corpuscular volume, MCH represents mean corpuscular hemoglobin, MCHC represents mean corpuscular hemoglobin concentration, RDW represents red blood cell distribution width, HGB represents hemoglobin, PLT represents platelet count, PCT represents plateletcrit, and MPV represents mean plasma volume. ${}^{2}$ Mean values ± standard errors (n=15). ${}^{a,b,c}$ represent significant differences among treatment groups at a confidence level of 95 % (P<0.05).

### 2.4 Determination of hematological parameters

Serum samples were thawed at $5{}^{\circ }$C and analyzed using an automated blood cell counter (VetScan I-STAT 1 handheld analyzer, Abaxis, USA) and a blood gas analyzer (RAPIDChem 744/754 blood gas analyzer, Simens, USA). Analyses were carried out within 1 h of sampling to determine the levels of hematocrit (HCT), red blood cells (RBC), mean corpuscular volume (MCV), mean corpuscular hemoglobin (MCH), mean corpuscular hemoglobin concentration (MCHC), red blood cell distribution width (RDW), hemoglobin (HGB), platelet (PLT), plateletcrit (PCT), mean plasma volume (MPV), electrolytes, blood pH, and blood gases of the birds to obtain their red blood cell and platelet profiles.

**Table 3:** Effect of different stocking densities on blood electrolytes (mmol L${}^{-1}$) in meat ducks exposed to heat stress.

	Treatment groups			
Items	CON	LSD	MSD	HSD
Sodium (Na${}^{+})$	136.7 ± 0.51${}^{*,c}$	152.3 ± 0.33${}^{a}$	151.6 ± 0.72${}^{a}$	147.6 ± 1.22${}^{b}$
Potassium (K${}^{+})$	3.15 ± 0.23${}^{c}$	4.58 ± 0.11${}^{a}$	4.64 ± 0.14${}^{a}$	4.10 ± 0.06${}^{b}$
Chloride (Cl${}^{-})$	132.7 ± 1.03${}^{c}$	113.7 ± 1.76${}^{a}$	114.7 ± 1.20${}^{a}$	106.7 ± 1.75${}^{b}$

${}^{*}$ Mean values ± standard errors (n=15). ${}^{a,b,c}$ represent significant differences among treatment groups at a confidence level of 95 % (P<0.05).

### 2.5 Serum immunoglobulin G and corticosterone

Levels of serum immunoglobulin G (IgG) and corticosterone were measured using a chicken ELISA kit (Bethyl Laboratories, Montgomery, TX, USA) and a corticosterone HS EIA kit (IDS, Ltd., Boldon, UK) in accordance with the protocols set by their manufacturers. The levels of these factors were calculated by measuring absorbances at 450 nm using a microplate reader (Molecular Devices Inc, New York, USA).

**Table 4:** Effect of different stocking densities on the blood pH and gas concentrations of meat ducks exposed to heat stress.

	Treatment groups			
Items${}^{1}$	CON	LSD	MSD	HSD
pH	7.08 ± 0.07${}^{2,c}$	7.35 ± 0.06${}^{b}$	7.37 ± 0.02${}^{b}$	7.46 ± 0.01${}^{a}$
p${\mathrm{CO}}_{2}$(mmHg)	32.83 ± 2.07${}^{c}$	45.32 ± 1.20${}^{a}$	44.57 ± 0.64${}^{a}$	39.30 ± 0.25${}^{b}$
${\mathrm{PO}}_{2}$(mmol L${}^{-1}$)	55.10 ± 1.29${}^{a}$	47.10 ± 0.94${}^{b}$	46.24 ± 0.59${}^{b}$	41.58 ± 0.82${}^{c}$
BEecf	-3.15 ± 0.09	-3.04 ± 0.09	-2.82 ± 0.14	-3.16 ± 0.45
${\mathrm{HCO}}_{3}$(mmol L${}^{-1}$)	18.13 ± 0.71${}^{c}$	24.56 ± 0.62${}^{a}$	24.61 ± 0.68${}^{a}$	22.46 ± 0.23${}^{b}$
TCO${}_{2}$(mmol L${}^{-1}$)	19.75 ± 0.77${}^{c}$	26.86 ± 0.61${}^{a}$	26.70 ± 0.77${}^{a}$	24.38 ± 0.49${}^{b}$

${}^{1}$ pH represents the hydrogen exponent, p${\mathrm{CO}}_{2}$ represents the partial pressure of carbon dioxide, ${\mathrm{PO}}_{2}$ represents the partial pressure of oxygen, BEecf represents the base excess in the extracellular fluid, ${\mathrm{HCO}}_{3}$ represents bicarbonate, T${\mathrm{CO}}_{2}$ represents total carbon dioxide. ${}^{2}$ Mean values ± standard errors (n=15). ${}^{a,b,c}$ represent significant differences among treatment groups at a confidence level of 95 % (P<0.05).

### 2.6 Animal behavioral traits

To determine the influence of HS on the daily behaviors of meat ducks, 24 meat ducks per treatment group were randomly selected and the behaviors of these birds were observed for 60 min each day under HS conditions (between 11:00 and 16:00). The control group was maintained under normal environmental heat conditions. Every movement of the chosen subject was monitored via video cameras (Camlife Image Recording Instruments V11.50, TianMin Products Science and Technology Development Co., LTD, Shenzhen, Guangdong, China) that were installed on the ceiling of the poultry house. The observations of the animal's behaviorial patterns were based on the scientific concept of animal behavioral traits from the UK Farm Animal Welfare Council (FAWC, 1995).

**Table 5:** Effect of different stocking densities on blood IgG and corticosterone levels (ng mL${}^{-1}$) in meat ducks exposed to heat stress.

	Treatment groups			
Items	CON	LSD	MSD	HSD
IgG	187.5 ± 4.01${}^{c}$	299.6 ± 5.63${}^{*,a}$	307.7 ± 3.38${}^{a}$	250.0 ± 3.03${}^{b}$
Corticosterone	28.71 ± 3.01${}^{c}$	61.33 ± 1.87${}^{b}$	62.96 ± 2.55${}^{b}$	91.42 ± 1.95${}^{a}$

${}^{*}$ Mean values ± standard errors (n=15). ${}^{a,b,c}$ represent significant differences among treatment groups at a confidence level of 95 % (P<0.05).

As a way of measuring the time and frequency of specific behaviors shown by the birds and evaluating their appearances, 10 min scan sampling was used per treatment group and a “deep skin harmful score” was obtained for each group. The animal's walking ability (gait score) (Kestin et al., 1992; Thomas et al., 2004), the presence of hock burns , drinking frequency, feather condition, and the presence of foot pad lesions were all measured using a three-point scale (1 represented normal, 2 represented mild, 3 represented severe) (Estevez et al., 2003; Škrbić et al., 2009; Zhu et al., 2012; Chen et al., 2015).

**Table 6:** Effect of stocking densities on the walking ability (gait), drinking frequency, presence of hock burn, presence of foot pad lesions, and feather condition scores in meat ducks exposed to heat stress.

	Treatment groups			
Behavioral traits	CON	LSD	MSD	HSD
Walking ability (gait)	1.00 ± 0.07${}^{*,c}$	1.38 ± 0.06${}^{b}$	1.84 ± 0.02${}^{b}$	2.92 ± 0.08${}^{a}$
Hock burns	1.00 ± 0.08${}^{c}$	1.15 ± 0.04${}^{b}$	1.86 ± 0.05${}^{b}$	2.78 ± 0.09${}^{a}$
Foot pad lesions	1.00 ± 0.05${}^{c}$	1.17 ± 0.07${}^{b}$	1.75 ± 0.04${}^{b}$	2.82 ± 0.05${}^{a}$
Feather condition	1.00 ± 0.16${}^{c}$	1.55 ± 0.03${}^{b}$	1.76 ± 0.08${}^{b}$	2.58 ± 0.07${}^{a}$
Drinking	1.00 ± 0.18${}^{d}$	1.37 ± 0.04${}^{c}$	2.07 ± 0.07${}^{b}$	3.00 ± 0.09${}^{a}$

${}^{*}$ Mean values ± standard errors (n=24). ${}^{a,b,c,d}$ represent significant differences among treatment groups at a confidence level of 95 % (P<0.05).

### 2.7 Statistical analysis

Data from this study– the main factors being heat stress and stocking density – were analyzed using a two-way ANOVA in SPSS (Statistical Package for Social Sciences; SPSS Inc., Chicago, IL, USA). Statistical significance was determined using a Duncan's multiple range test at a confidence level of 95 % (P<0.05) (SPSS, 2010).

## 3 Results

Lipid profiles and levels of glucose, ALT, and AST are shown in Table 1. The concentrations of blood TAG, TC, LDL-C, AST, and ALT were found to be higher in the HS groups compared with the CON group (P<0.05): they were the highest in HSD group, but similar between the LSD and MSD groups. In contrast, the concentrations of HDL-C and glucose were lower in the HSD group compared to the other groups (P<0.05). The glucose concentration was also lower in the MSD group compared with the CON group. AST and ALT concentrations were higher in the HS groups compared with the CON group (P<0.05); concentrations of AST and ALT were higher in the HSD group than in the LSD or MSD groups (P<0.05), but were not different between LSD and MSD.

Red blood cell (RBC) and platelet (PLT) profiles are shown in Table 2. The concentrations of blood HCT, RBC, MCV, MCH, MCHC, RDW, HGB, PLT, PCT, and MPV were lower in the HS groups compared with the CON group, with HSD showing the lowest levels of these factors (P<0.05); however, the levels were similar between the LSD and MSD groups.

Concentrations of blood electrolytes are shown in Table 3. These values were higher in the HS groups compared with the CON group, with HSD displaying the highest levels (P<0.05); however, the levels were similar between LSD and MSD.

Results of the blood pH values and gas concentrations are summarized in Table 4. Blood pH values were higher in the HS groups compared with the CON group(P <0.05), but were similar between the LSD and MSD groups. Furthermore, the concentrations of blood p${\mathrm{CO}}_{2}$, ${\mathrm{HCO}}_{3}$, and T${\mathrm{CO}}_{2}$ were higher in the HS groups compared with the CON group (P<0.05). They were also higher in HSD than in LSD or MSD (P<0.05). However, they were found to be similar between LSD and MSD. Conversely, concentrations of p$O_{2}$ were higher in the CON group compared with the HS groups (P<0.05), but were similar between the LSD and MSD groups.

Blood IgG and corticosterone concentrations are shown in Table 5. The concentrations of blood IgG and corticosterone were significantly higher in the HS groups (P<0.05) compared with the CON group. They were also significantly higher in HSD (P<0.05) than in LSD or MSD, but they were similar between LSD and MSD.

Scores of animal behavioral traits under HS are shown in Table 6. Damage scores regarding the walking ability (gait), drinking frequency, presence of foot pad dermatitis, presence of hock burns, and feather condition of the HS groups were significantly higher (P<0.05) than those of the CON group. They were also significantly higher (P<0.05) in the HSD group than in the LSD or MSD groups. However, they were similar between LSD and MSD except for the fact that the drinking score was higher in MSD than in LSD.

## 4 Discussion

The findings of this study are similar to prior literature which shows that the serum levels of ALT, AST, glucose, TC, TAG, and LDL-C in broiler chickens exposed to high ambient temperature are higher, while HDL-C levels are higher in the thermoneutral zone group (chickens reared at constant room temperature) (Chand et al., 2018). Furthermore, other reports show that the levels of blood TC, LDL-C, and AST in broiler chickens in a high-temperature environment are higher, while the levels of AG, HDL-C, glucose, and ALT are lower than those found in the control group (Bueno et al., 2017). The body of an animal can respond to stressors to maintain homeostasis; however, when homeostasis is disturbed by HS and HSD animals will try to develop a response mechanism involving physiological and behavioral adjustments to adapt themselves to the disturbance (Bueno et al., 2017). AST levels in the HSD group under HS were significantly increased. This could potentially lead to hepatic or muscular disorder and serious liver damage (Abudabos et al., 2013; Capitelli and Crosta, 2013).

Some studies have reported that ducks exposed to HS and HSD have lower levels of certain hematological parameters compared to control ducks due to the damage to red blood cells, the reduction in the output of red cells, or the decrease in the number and size of red blood cells (Bueno et al., 2017; Park and Park, 2017). Under normal environmental conditions, a decrease of red blood cells and haemoglobin levels in humans or animals can cause iron-deficiency anemia. Ducks under HS and HSD suffer from hemodilution due to increased water absorption even though there is no change in plasma volume. This can lead to the evaporation of water from cells, and can, in turn, lower the number of red blood cells and the haemoglobin level (Turkyilmaz, 2008; Park and Kim, 2017).

The sodium–potassium ATP pump is critical for maintaining the metabolism of biomass energy and the balance of water in the cell. The concentrations or densities of sodium, potassium, and chloride in the blood of poultry and buffalo cattle are known to be reduced due to HS (Kumar et al., 2010). Under HS and HSD conditions, panting by poultry can increase the loss of potassium via fecal urine, and this disruption the blood electrolyte balance can potentially lower productivity (Borges et al., 2004; Park and Park, 2017). When birds are exposed to HS and HSD, their body temperatures increase. In addition, as previously stated, hemodilution occurs and potassium is emitted in excess; thus, the concentration of potassium in the blood is decreased. The decrease of the blood chloride level in birds exposed to HS and HSD means that more chlorides are needed to accelerate oxidization in body fluids in order to normalize the blood pH, which is rapidly escalated by blood alkalosis (Park and Kim, 2016; Park and Park, 2017).

Our results have shown that HSD rearing increases the blood pH values of meat ducks exposed to HS in addition to lowering their blood gas concentrations; this could eventually induce blood alkalosis, which is known to cause problems in the central nervous systems of bird (Borges et al., 2007; Park and Park, 2017). Birds kept under HS and HSD conditions will try to lower blood p${\mathrm{CO}}_{2}$ to bring their body temperatures down. This is done by removing ${\mathrm{CO}}_{2}$ from their lungs via panting (Mahmoudnia and Madani, 2012; Park and Kim, 2017).

Under stress, birds show higher concentrations of corticosterone while concentrations of their immunity substances are found to be lower than the control group (Fraisse and Cockrem, 2006). HSD rearing of meat ducks exposed to HS also causes reduced serum immune protein and IgG concentrations, while concentrations of corticosterone, a stress hormone, are increased, which leads to increased mortality.

This study assumes that the HSD rearing of meat ducks during summertime will stress the poultry and affect their behavior. The gait score is a subjective evaluation of the animal's walking ability caused by the ducks' physical inactivity due to the shortage of space when stocking densities are increased (Škrbić et al., 2009). An inverse correlation between the stocking density and drinking frequency per duck was observed in this study. This research also found that the number of times that ducks in HSD group drank was notably lower compared with animals in the LSD or MSD group. Animals that drink more water under HS and HSD rearing conditions show natural drinking and water-associated activities, such as straining and preening their plumage with water, which are considered to be behavioral requirements that satisfy “healthy” animal behavioral traits (Heyn et al., 2006). In meat ducks exposed to HS, the need to drink increases as the stocking density is increased. When litter becomes dirty or the lying resting time of animals is increased, the frequency of the occurrence of hock burns and foot pad dermatitis is increased. In the event of high-density rearing, hock burns, also known as ammonia burns, occur due to a combination of moisture and the high ammonia content in the litter (Berg, 2004). Hock burns and foot pad dermatitis are closely related to one another, and litter quality is critical factor controlling the occurrence of such health issues (Dawkins et al., 2004; Kiaer et al., 2006). Under HS and HSD rearing conditions, ducks show poor quality feathers, which can lead to different skin lesions (Škrbić et al., 2009). The negatively affected behavioral traits of ducks under HS and HSD conditions also make it difficult for them to take in the diet provided, which results in a decrease in body weight gain and an increase in mortality due to poor nutritional metabolism and unbalanced blood homeostasis.

## 5 Conclusions

The findings of this study show that when meat ducks are exposed to heat stress in combination with high stocking density conditions, the blood homeostasis of the poultry is decreased due to environmental stress which lowers red blood cell and platelet profiles, blood electrolytes, blood gas, and IgG concentrations but increases concentrations of ALT, AST, pH, and corticosterone. Rearing meat ducks at a stocking density of three to four birds per square meter (m${}^{2}$) during summer was found have an obvious influence on blood homeostasis and the behavioral traits of the animals, putting them in a dangerous position.

## Data Availability

The data sets are available upon request from the corresponding author.
